# Development of Peptide-Based Lineage-Specific Serology for Chronic Chagas Disease: Geographical and Clinical Distribution of Epitope Recognition

**DOI:** 10.1371/journal.pntd.0002892

**Published:** 2014-05-22

**Authors:** Tapan Bhattacharyya, Andrew K. Falconar, Alejandro O. Luquetti, Jaime A. Costales, Mario J. Grijalva, Michael D. Lewis, Louisa A. Messenger, Trang T. Tran, Juan-David Ramirez, Felipe Guhl, Hernan J. Carrasco, Patricio Diosque, Lineth Garcia, Sergey V. Litvinov, Michael A. Miles

**Affiliations:** 1 Faculty of Infectious and Tropical Diseases, London School of Hygiene and Tropical Medicine, London, United Kingdom; 2 Departamento de Medicina, Universidad del Norte, Barranquilla, Colombia; 3 Laboratorio de Chagas, Hospital das Clinicas, Universidade Federal de Goiás, Goiânia, Goias, Brazil; 4 Centro de Investigación en Enfermedades Infecciosas, Escuela de Biología, Pontificia Universidad Católica del Ecuador, Quito, Ecuador; 5 Tropical Disease Institute, Department of Biomedical Sciences, Heritage College of Osteopathic Medicine, Ohio University, Athens, Ohio, United States of America; 6 Facultad de Ciencias Naturales y Matemáticas, Universidad del Rosario, Bogotá, Colombia; 7 Centro de Investigaciones en Microbiología y Parasitología Tropical, Universidad de los Andes, Bogotá, Colombia; 8 Universidad Central de Venezuela Instituto de Medicina Tropical, Caracas, Venezuela; 9 Unidad de Epidemiología Molecular, Instituto de Patología Experimental, Consejo Nacional de Investigaciones Científicas y Técnicas (CONICET), Universidad Nacional de Salta, Salta, Argentina; 10 Facultad de Medicina, Universidad Mayor de San Simón, Cochabamba, Bolivia; 11 Aptum Biologics Ltd, Southampton, United Kingdom; US Food and Drug Administration, United States of America

## Abstract

**Background:**

Chagas disease, caused by infection with the protozoan *Trypanosoma cruzi*, remains a serious public health issue in Latin America. Genetically diverse, the species is sub-divided into six lineages, known as TcI–TcVI, which have disparate geographical and ecological distributions. TcII, TcV, and TcVI are associated with severe human disease in the Southern Cone countries, whereas TcI is associated with cardiomyopathy north of the Amazon. *T. cruzi* persists as a chronic infection, with cardiac and/or gastrointestinal symptoms developing years or decades after initial infection. Identifying an individual's history of *T. cruzi* lineage infection directly by genotyping of the parasite is complicated by the low parasitaemia and sequestration in the host tissues.

**Methodology/Principal Findings:**

We have applied here serology against lineage-specific epitopes of the *T. cruzi* surface antigen TSSA, as an indirect approach to allow identification of infecting lineage. Chagasic sera from chronic patients from a range of endemic countries were tested by ELISA against synthetic peptides representing lineage-specific TSSA epitopes bound to avidin-coated ELISA plates via a biotin labelled polyethylene glycol-glycine spacer to increase rotation and ensure each amino acid side chain could freely interact with their antibodies. 79/113 (70%) of samples from Brazil, Bolivia, and Argentina recognised the TSSA epitope common to lineages TcII/TcV/TcVI. Comparison with clinical information showed that a higher proportion of Brazilian TSSApep-II/V/VI responders had ECG abnormalities than non-responders (38% vs 17%; p<0.0001). Among northern chagasic sera 4/20 (20%) from Ecuador reacted with this peptide; 1/12 Venezuelan and 1/34 Colombian samples reacted with TSSApep-IV. In addition, a proposed TcI-specific epitope, described elsewhere, was demonstrated here to be highly conserved across lineages and therefore not applicable to lineage-specific serology.

**Conclusions/Significance:**

These results demonstrate the considerable potential for synthetic peptide serology to investigate the infection history of individuals, geographical and clinical associations of *T. cruzi* lineages.

## Author Summary

Chagas disease remains a significant public health issue in Latin America. Caused by the single-celled parasite *Trypanosoma cruzi*, the main route of infection is via contact with contaminated faeces from blood-sucking triatomine bugs, but following successful insecticide spraying campaigns, congenital, food-borne, and transfusion/transplantation routes of infection have become more relevant. In the absence of successful chemotherapy, *T. cruzi* usually persists in the body for life, and in symptomatic cases may lead to death or debilitation by heart failure and/or gastrointestinal megasyndromes. As a species, *T. cruzi* displays great genetic diversity, and is subdivided into lineages called TcI - TcVI. Associating *T. cruzi* lineage with clinical symptoms is a key goal of Chagas disease research. Direct isolation and typing of *T. cruzi* from chronically infected patients is hampered by the sequestration of the parasite in host tissues. Identifying lineage-specific antibodies in serum provides an alternative approach to determining an individual's history of infection. Here, we performed lineage-specific serology using samples from a range of South American countries. We show that lineage-specific seropositivity is associated with geographical distributions and clinical outcome. These findings have wide implications for further diagnostics development and improved understanding of the epidemiology of Chagas disease.

## Introduction

Chagas disease (South American trypanosomiasis) is still considered to be the most important parasitic disease in Latin America, despite notable success with control of household infestation by the triatomine insect vectors. Up to 8 million people are estimated to be chronically infected with the causative agent *Trypanosoma cruzi*, of whom at least 30% are likely to develop chagasic cardiomyopathy, in some cases with megasyndromes of the intestinal tract [1], [2]. Vector borne transmission is usually by contamination of mucous membranes or abraded skin with *T. cruzi* infected triatomine faeces and sporadic oral outbreaks occur due to triatomine contamination of food [3]. Infection can also be propagated by congenital transmission and blood or organ donation, and this may arise among migrant populations far beyond the endemic regions in Latin America [4].

The species *T. cruzi* is remarkably diverse genetically and is currently described as comprising six distinct lineages or discrete typing units (DTUs, TcI-TcVI) [5]. The six lineages have complex disparate but partially overlapping geographical and ecological distributions and are circumstantially associated with different epidemiological features [6], [7]. TcI is the principal agent North of the Amazon, in association with chagasic heart disease but where megasyndromes are considered to be rare. TcII is one of three principal agents of Chagas disease in the Southern Cone region of South America, where chagasic cardiomyopathy, megaoesophagus and megacolon are found. TcIII is seldom isolated from humans but is widely distributed with the natural armadillo host *Dasypus novemcinctus*. TcIV is a sporadic secondary agent of Chagas disease in Venezuela [8]. TcV and TcVI, like TcII, are also agents of Chagas in the Southern Cone region, and are known to be relatively recent hybrids of TcII and TcIII [7], [9].

Parasitological diagnosis in the acute phase of *T. cruzi* infection is by microscopy of fresh blood films, thin blood films, thick blood films or by haematocrit centrifugation and examination of the buffy coat, the latter being recommended particularly for congenital cases. In the chronic phase recovery of live organisms may be attempted by multiple blood cultures or xenodiagnosis with colony bred triatomine bugs but with limited sensitivities, or parasite DNA may be detectable by amplification.

Serological diagnosis of *T. cruzi* infection is usually performed by either indirect immunofluorescence (IFAT) or indirect haemaglutination (IHA) or enzyme-linked immunosorbent assay (ELISA), giving >94% sensitivity and specificity [2]. There are several commercially available diagnostic kits, including rapid lateral flow tests but sensitivities may not be equivalent, particularly when they are used in regions where non-homologous genetic lineages of *T. cruzi* are prevalent [8]–[10]. These serological methods give no information on the genetic lineage or lineages that a patient carries, and are not designed for that purpose.

A key objective of Chagas research therefore remains to follow up in detail the circumstantial evidence of a relationship between infecting *T. cruzi* lineage and the clinical outcome [6], [7], [11]. However, such analysis is complex and vulnerable to multiple confounders, including diversity of host susceptibility. Even if *T. cruzi* isolates can be recovered from the infected blood by parasitological diagnosis or if DNA can be amplified from blood, genotyping methods [12], [13] do not provide an entire profile of the infecting lineages in an individual patient, because distinct *T. cruzi* lineages may be sequestered in the tissues [14]. An approach to overcoming this limitation is to identify infecting *T. cruzi* lineage in a more indirect way. One strategy to achieve this is by serological detection of antibodies that are produced in response to lineage-specific antigens.

Di Noia et al [15] described the trypomastigote small surface antigen (TSSA), encoded by a member of the *TcMUCIII* mucin gene family, expressed on the mammalian bloodstream trypomastigote stage of the *T. cruzi* life cycle. The authors reported that TSSA is dimorphic in sequence, with TSSA-I being present in TcI, and TSSA-II found in TcII-TcVI. On the basis of this finding the authors pioneered lineage-specific serology for Chagas disease through application of a TSSA-II recombinant antigen to serology with patients from the Southern Cone region of South America. Chagasic patients were only TSSA-II seropositive, which led to the suggestion that TcI could be benign. However, this suggestion was in conflict with the geographical predominance of TcI North of the Amazon and the acute and chronic clinical presentations of known TcI infections [16], [17]. In subsequent publications *E. coli*-produced recombinant TSSA proteins have been used more widely for serology with humans and animals [18]–[23].

We have previously analysed *TSSA* diversity among a panel of *T. cruzi* isolates representing a broad geographical and ecological range of lineages TcI-TcVI [24]. We found a greater lineage-specific diversity than had previously been described. Lineages TcII, TcV, and TcVI were shown to share a common TSSA sequence. However, in both of the hybrid lineages TcV and TcVI we found that two TSSA alleles were present at an heterozygous locus within the polymorphic epitope: one haplotype was shared with TcII and in the second haplotype a Thr was replaced by Ala at position 44 of the protein. Lineage-specific TSSA sites were also found in TcIII and TcIV strains [24]. Cánepa et al [25] suggested a functional significance for this diversity in that the TcII/TcV/TcVI form of TSSA, but not the TcI form, has the property of binding surface receptor(s) and inducing signalling pathways in host cells prior to parasite internalisation.

Recently, Mendes et al [26] used a bioinformatic analysis of the reference genome of the TcVI strain CL Brener [27] to identify candidate peptides for differential screening with sera from mice experimentally infected with single, known *T. cruzi* lineages. A resultant peptide, derived from a putative RNA-binding protein, was reported to be applicable for TcI serology [26].

Here, we have used our expanded knowledge of the range of TSSA diversity to design and synthesise lineage-specific peptides. We assess the capacity of these peptides to provide antigens for lineage-specific serology by ELISA and thus reveal which lineages have infected individual patients during their lifetime. Furthermore, we examine the geographical and clinical distribution of recognition of the synthetic peptide epitopes. In addition, we also investigate the diversity of the gene coding for the peptide described [26] as applicable for TcI-specific serology.

## Materials and Methods

### Ethics statement

Human sera were collected as part of routine diagnostic examination, with local institutional ethical approvals, and in accord with EC ethical standards established as part of the ChagasEpiNet international collaboration. All human sera were anonymised and coded by letters and numbers that did not reveal patient identities. Production of mouse sera adhered to the European 3Rs policy of Refinement, Reduction and Replacement (99/167/EG: Council decision of 25/1/99), took place in authorised animal facilities by licensed staff in agreement with the European Directive 86/609/EEC, and with review and approvals under UK Home office regulations [Animals (Scientific Procedures) Act 1986; project licence number 70/6997 to the London School of Hygiene and Tropical Medicine].

### Mouse and human sera

Mouse sera were from mice previously inoculated intraperitoneally with 10^6^ organisms from stationary phase cultures containing infective metacyclic trypomastigotes, of known biological clones of *T. cruzi* representing the lineages. Sera were separated from whole mouse blood by allowing clotting at room temperature, overnight storage at 4°C, centrifugation at 12000×*g* for 10 mins and removal of the supernatant serum. Serum samples were stored 1∶1 with glycerol at −20°C.

Human sera were from chronic cases of Chagas disease, confirmed by a combination of parasitological and serological diagnosis. As shown in Table 1, 113 samples were from the Southern Cone countries, Brazil, Bolivia and Argentina, and 66 samples were from countries North of the Amazon, Colombia, Ecuador, Venezuela, where TcI has been considered to predominate. Brazilian sera were from patients who had a positive parasitological diagnosis at the time of serum collection, together with a full clinical history, their geographical origin, age and sex. Institutes providing sera were: Hospital das Clinicas, Goiânia, Brazil; Universidad Mayor de San Simon, Cochabamba, Bolivia; Universidad Nacional de Salta, Argentina; Universidad Central de Venezuela, Caracas, Venezuela; Universidad de los Andes, Bogotá, Colombia; Pontificia Universidad Católica del Ecuador, Quito, Ecuador. Endemic healthy controls were from the Hospital das Clinicas, Goiânia, Brazil, and additional controls were 17 sera from Colombia that were serologically negative to *T. cruzi* lysate.

**Table 1:** Geographical distribution of antibody responses to lineage-specific synthetic peptides, as determined by ELISA.

		TSSA peptide reaction								
		Lineage-specific						Chimera		Non-specific^g^
	n	I	II/V/VI	III	IV	V/VI	Non-reactive	I/II	II/I	
**Brazil**	98^a^	1^b^	67	1^b^	1^b^	11/67 of TcII/V/VI	28	9/67 of TcII/V/VI^c^	55/67 of TcII/V/VI	2
**Bolivia**	10	0	9	0	0	1/9 of TcII/V/VI	0	0/9 of TcII/V/VI	9/9 of TcII/V/VI	1
**Argentina**	5	1^d^	3	1^d^	1^d^	0/3 of TcII/V/VI	1	0/3 of TcII/V/VI	3/3 of TcII/V/VI	0
Colombia*	34	0	0	1^e^	1^e^	0	33	ND	ND	0
**Ecuador**	20	0	4	0	0	1/4 of TcII/V/VI	16	2/4 of TcII/V/VI^c^	4/4 of TcII/V/VI	0
**Venezuela**	12	0	0	1^f^	1^f^	0	10	1/1^f^	0	1
**EHC (Brazil)**	7	0	0	0	0	0	7	0	0	0
**TOTAL**	**186**	**2**	**83**	**4** h	**4** h	**13 of 83 of all TcII/V/VI**	**95**	**11 of the 83 TcII/V/VI positives & 1 of the 4 TcIV positives**	**71 of the 83 TcII/V/VI positives**	**4**

EHC =  Endemic healthy controls (* a further 17 Colombian sera that were serologically negative with the lysate were included in the peptide ELISAs as additional controls); ND =  not determined.

a these 98 comprised 1 sample from each of 90 patients, plus 2 paired samples from each of 4 patients. All eight paired samples reacted with TSSApep-II/V/VI, and are included within the 67 Brazilian reactors to this peptide. 1 set of these pairs also reacted with TSSApep-V/VI.

b same sample, which did not react with TSSApep-II/V/VI, TSSApep-V/VI or chimeras.

c these 9 samples also reacted with chimera TSSA-II/-I peptide.

d same sample, which did not react with TSSApep-II/V/VI, TSSApep-V/VI or chimeras.

e same sample.

f same sample, which did not react with TSSApep-I, TSSApep-II/V/VI, TSSApep-V/VI or chimera TSSApep-II/-I.

g non-specific binding; see text.

h in each case the same sample reacted with TSSApep-III and TSSApep-IV.

### Synthesis of lineage-specific peptides

The synthetic peptides were prepared with an amino terminal biotin molecule linked via a polyethylene glycol-glycine spacer so that they could be bound to avidin-coated ELISA plates. Importantly, this method increased their rotation and ensured that each amino acid side chain could freely interact with antibodies, as opposed to being adsorbed onto the solid phase where some amino acid side chains would be unavailable, as discussed previously [28].

**Figure 1:**
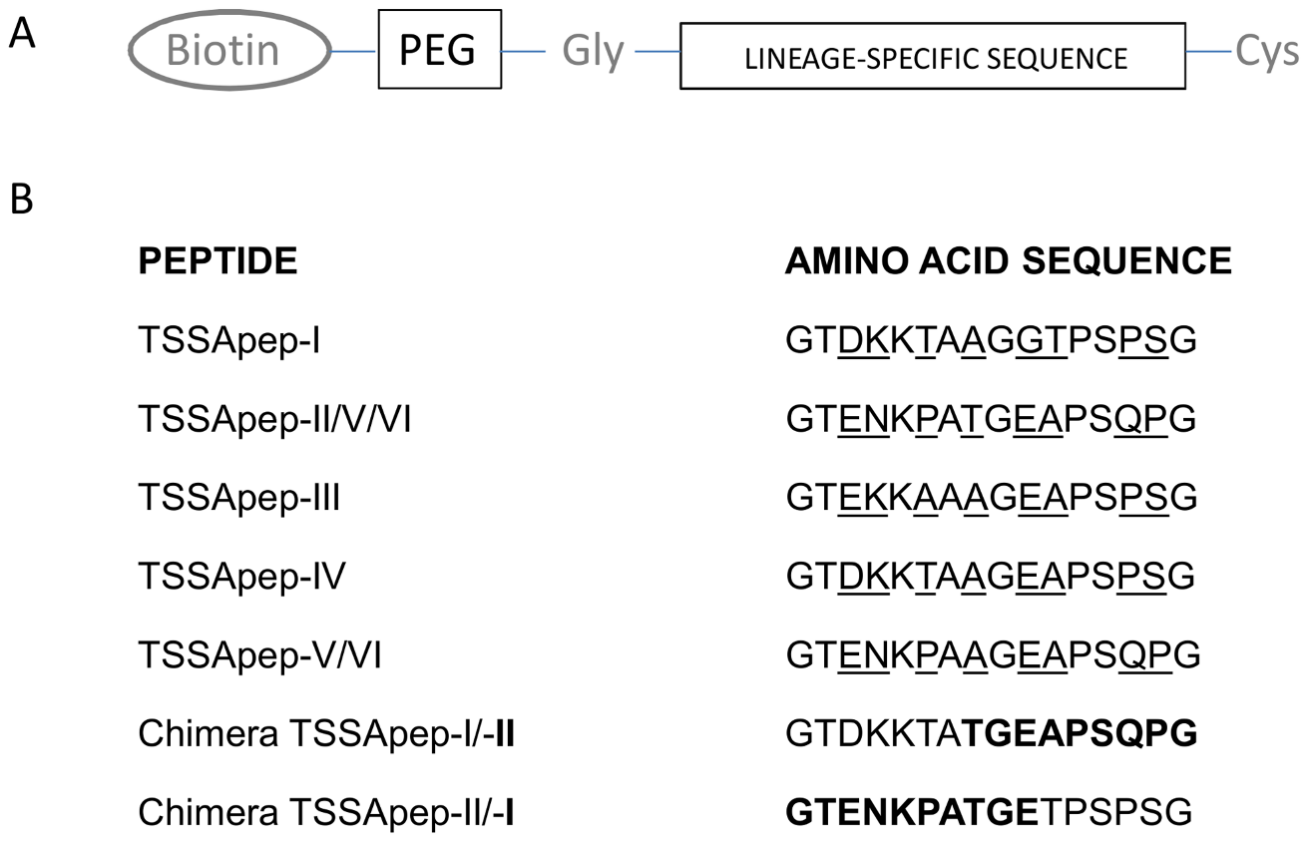
TSSA provides potential epitopes that are *T. cruzi* lineage-specific. [A] Components of the peptides synthesised: N-terminal biotinylation; PEG spacer; Gly; the lineage-specific sequence; C-terminal Cys. [B] Amino acid sequences of the *T. cruzi* lineage-specific TSSA potential epitopes in the synthetic peptides (TSSApep-), with polymorphic residues underlined; for the two chimeric peptides the TSSA-II residues are shown in bold.

Design of the peptides was based on the *T. cruzi* TSSA lineage-specific amino acid sequences previously described [24]; chimeric peptides comprised by TSSA-I and TSSA-II sequences were also designed and synthesised (Results; Figure 1). Synthetic peptides were prepared at the 20 µM scale on 100–200 mesh-size Fmoc-Cys(Trt) Wang resin (0.5 mmol/g) (856006: Novabiochem, UK) using a Zinsser Analytic SMPS 350 (Zinsser Analytic, UK) or Advanced Chemtech Apex 396 (Advanced Chemtech, USA) robotic multiple peptide synthesizer. Aspartamide formation of aspartic acid residues was reduced by the use of OMpe-protected Fmoc-Asp(OMpe)-OH (852104: Novabiochem, UK). The coupling steps were performed using 0.5 M Fmoc-protected amino acids diluted in 6.76% (wt/vol) 1-hydroxybenzotriazole (HOBt)/dimethylformamide (DMF) (Activotec, UK/Rathburn Chemicals Ltd., UK) activated using 0.5 M N,N,N′,N′-tetramethyl-O-(1H-benzotriazol-1-yl)uranium hexafluorophosphate (HBTU) (851006: Novabiochem, UK) with 1M *N*,*N*-diisopropylethylamine (DIPEA) (Rathburn Chemicals Ltd., UK), while the deprotection steps were performed using 20% (vol/vol) piperidine/DMF (Rathburn Chemicals Ltd., UK). The carboxyl- and amino- regions flanking the core epitope sequences contained additional glycine (G) residues to increase rotation (high dihedral (ψ against φ) angles) of their carboxyl-terminal cysteine (C) residue and their amino-terminal spacer and molecular label. Their amino termini were labelled via a polyethylene glycol (PEG) spacer (Fig 1A) through sequential couplings with 0.5 M Fmoc-NH-(PEG)_2_-COOH (13 atoms or 20 atoms) (851034 or 851031: Novabiochem, UK) followed by 0.5 M biotin (B4501: Sigma Aldrich, UK) using the more efficient coupling agent, 0.5 M N,N,N′,N′-tetramethyl-O-(7-azabenzotriazol-1-yl)uranium hexafluorophosphate (HATU) (851013: Novabiochem, UK) containing 1 M DIPEA. The final peptides were each washed 5 times with dichloromethane and then methanol (Rathburn Chemicals Ltd., UK) before being dried in a freeze-drier (Edwards, UK). Peptide cleavage was performed by reaction for 3–4 hours using 1% (wt/vol) phenol, 2% H_2_O, 2.5% (vol/vol) triisopropyl silane (233781: Sigma Aldrich, UK) and 2% (vol/vol) 2,2′ (ethylenedioxy) diethanethiol (3,6-dioxa-1,8-octanedithiol (DODT)) (465178: Sigma Aldrich, UK) in trifluoroacetic acid (Rathburn Chemicals Ltd., UK) [29]. The cleaved peptides were then precipitated in cold (0°C) peroxide-free diethyl ether (Rathburn Chemicals Ltd., UK), centrifuged at 2,000×g; the supernatants were discarded and the precipitation and centrifugation steps were repeated twice. The peptides were then dried under a stream of anhydrous argon gas (BOC, UK) before being stored at −80°C.

For purification each peptide was subsequently dissolved in 500 µl of 2,2,2, trifluoroethanol (T63002: Sigma Aldrich, USA), made to 5.5 ml with 5% (vol/vol) far UV grade acetonitrile (Rathburn Chemicals Ltd., UK) in H_2_O containing 0.1% (vol/vol) trifluoroacetic acid, and then subjected to reversed-phase HPLC using a 5 ml injection loop, a 5–95% acetonitrile gradient run at 9 ml/min over 30 min through an ACE C18–300 Å 250×21.2 mm preparative column (ACE-231-2520: Advanced Chromatography Technologies, UK) in a Beckman Gold preparative HPLC system (Beckman, USA). The main peaks, detected at a wavelength of 215 nm, were collected and freeze-dried before storage at −80°C.

Additionally, peptides TSSApep-II/V/VI, chimera TSSApep-I/-II, and MenTcI were also synthesised commercially (Genosphere Biotechnologies, Paris, France).

Purified peptides were prepared as 1 mg/ml stock solutions in PBS and the addition of biotin in the last coupling was assessed by ELISA. For this assay, 10 µg/ml of each peptide was prepared in 1× carbonate-bicarbonate coating buffer (15 mM Na_2_CO_3_, 34 mM NaHCO_3_, pH 9.6) and added at 50 µl/well to 96-well ELISA plates (735–0465: Immulon 4HBX, VWR, UK). Plates were covered with an adhesive sheet and incubated overnight at 4°C. Following three washes with PBS containing 0.05% (vol/vol) Tween 20 (P7949: Sigma Aldrich, UK) (PBS/T), 200 µl/well blocking buffer (PBS/2% skimmed milk powder (Premier International Foods, Spalding, UK) was added and incubated at 37°C for 2 hrs. Following three washes, a 1∶2000 dilution of peroxidase-labeled streptavidin (S2438: Sigma Aldrich, UK) in PBS/T containing 2% skimmed milk powder (PBS/T/M) was added at 50 µl/well, and incubated at 37°C for 1 hour. After washing six times with PBS/T, 50 mM phosphate/citrate buffer (pH 5.0) containing 2 mM *o*-phenylenediamine HCl (P1526: Sigma Aldrich, UK) and 0.005% (vol/vol) H_2_O_2_ (216763: Sigma Aldrich, UK) was added at 50 µl/well and the plates were incubated in the dark at room temperature for 10 minutes. The substrate reactions were then stopped by the addition of 2M H_2_SO_4_ (25 µl/well) and the absorbance values were determined at a wavelength of 490 nm (MRX, Dynatech, USA).

### Production of whole-cell lysate antigen

*T. cruzi* was cultured as previously described [30]. For production of lysate antigen, mid-to-late log phase cultures of a TcII strain (IINF/PY/00/Chaco23cl4) of *T. cruzi* were centrifuged at 800×*g* for 10 mins at 4°C in an Allegra X-15R benchtop centrifuge (Beckman Coulter, UK). After washing in PBS, cell pellets were subjected to 3 cycles of flash-freezing in liquid nitrogen and thawing in a cold water bath. Cell lysates were then sonicated for 3×30 sec, with intervals on ice, using a Soniprep 150 sonicator (MSE), at 12 µ amplitude. Sonicated lysates were centrifuged at 13000 rpm for 1 min, and the supernatant used as antigen in ELISA. Protein concentration was determined using the BCA Protein Assay kit (PN23227: Fisher Scientific, UK).

### Lineage-specific peptide ELISA

Immulon 4HBX 96-well flat bottomed ELISA plates were coated with 1 µg/100 µl/well of avidin (A9275: Sigma, UK) diluted in 1× carbonate-bicarbonate coating buffer for binding to lineage-specific peptide, and in separate wells coating was with TcII *T. cruzi* lysate at 0.2 µg/100 µl/well to act as a serologically positive control for each sample. Plates were covered with an adhesive sheet and incubated overnight at 4°C. The following day, unbound avidin and lysate were removed, the plate washed three times with wash buffer PBS/T, then wells were blocked with 200 µl blocking buffer PBS/T/M at 37°C for 2 hrs. Following three washes, 1 µg/100 µl/well TSSA lineage-specific peptide in PBS/T/M was incubated with the avidin-coated wells at 37°C for 1 hr. Following three washes, 100 µl/well of a 1∶200 dilution of serum in PBS/T/M was added and incubated at 37°C for 1 hr. Following six washes, 100 µl/well of donkey anti-human IgG (H+L)-HRP (709-035–149: Jackson Immunoresearch, Pennsylvania, USA), diluted 1∶5,000 in PBS/T/M was added, and incubated at 37°C for 1 hr. Following six washes, plates were developed and read as described above, except that the volumes were 100 µl for substrate and 50 µl for 2M H_2_SO_4_. Replica plates were run in duplicate simultaneously.

### Statistical analysis

Cut-off values for ELISAs with human sera and peptides were calculated from the mean plus 3 standard deviations compared to the endemic healthy controls from Goiânia, Brazil. Statistical analysis (2-tailed unpaired t-test) on the Brazilian TSSApep-II/V/VI seropositives and non-responders was performed using GraphPad Prism version 4.02 for Windows (GraphPad Software, San Diego, California, USA).

### Analysing diversity of the putative TcI epitope

We designed PCR primers MenTcI FOR (5′ ATGCCACAATCGAAACCAAG 3′) and MenTcI REV (5′ TCACAACAAACGTTTGGCTG 3′) (synthesised by Eurofins MWG Operon, Germany) to amplify the whole open reading frame (ORF) of the putative RNA–binding protein (Tc00.1047053511837.129) which was described as containing an epitope and corresponding peptide applicable for TcI serology [26]. *T. cruzi* strains, from which genomic DNA was used as amplification template, are listed in Table 2. Amplification reactions were performed in a total volume of 20 µl, and comprised of 1×NH_4_ reaction buffer supplemented with 1.5 mM MgCl_2_ (Bioline, UK), 200 mM dNTPs (New England Biolabs, UK), 10 pmol of each primer, and 1 U BioTaq DNA polymerase (Bioline). Amplification conditions were: 1 cycle of 94°C, 3 mins; 25 cycles of 94°C for 30 secs, 55°C for 30 secs, 72°C for 30 secs; 1 cycle of 72°C for 10 mins. Five microliters of the PCR reaction were analysed by electrophoresis on 1.5% agarose gels (Bioline); amplification products were purified from the remaining reaction by precipitation with an equal volume of isopropanol at room temperature, followed by washing with 70% EtOH, air-drying and resuspension in ddH_2_O. Bi-directional DNA sequencing, using each PCR primer separately at 3.2 pmol, was achieved using a BigDye Terminator v3.1 RR-100 kit (Applied Biosystems, UK) according to standard protocols. Sequence alignment was performed using BioEdit software [31]. In parallel, the coding region of the TSSA gene containing lineage-specific sequences was also sequenced, as described previously [24], to confirm lineage identity.

**Table 2:** *T. cruzi* strains used here for comparative analysis of the ORF containing the reported TcI-applicable peptide (GenBank accession numbers refer to sequences determined here).

Lineage	Strain	Origin	Host/vector	Tc00.1047053511837.129 [26] ^a^	GenBank
				E N S A N P P P P D R S L P T P	
TcI	MHOM/BR/78/Sylvio-X10/1	Belém, Brazil	*Homo sapiens*	. . . . . . . . . . . . . . . .	KJ395471
	MHOM/PE/00/SaxP18	Majes, Peru	*Homo sapiens*	. . . . . . . . . . . . . . . .	KJ395472
	MPHI/BO/00/SJM41	Beni, Bolivia	*Philander opossum*	. . . . . . . . . . . . . . . .	KJ395473
	MDID/BO/00/SMA2	Beni, Bolivia	*Didelphis marsupialis*	. . . . . . . . . . . . . . . .	KJ395474
	MDID/BO/00/SJM37	Beni, Bolivia	*Didelphis marsupialis*	. . . . . . . . . . . . . . . .	KJ395475
	MPHT/BO/00/COTMA47	Cotopachi, Bolivia	*Phyllotis ocilae*	. . . . . . . . . . . . . . . .	KJ395476
TcII	MHOM/CL/00/IVV	Cuncumen, Chile	*Homo sapiens*	. . . . . . . . . . . . . . . .	KJ395477
	MHOM/BR/00/Y	São Paulo, Brazil	*Homo sapiens*	. . . . . . . . . . . . . . ^S^_T_ .	KJ395478
	MHOM/CL/00/CBB	Region IV, Chile	*Homo sapiens*	. . . . . . . . . . . . . . ^S^_T_ .	KJ395479
	IINF/BO/00/Tu18	Tupiza, Bolivia	*Triatoma infestans*	. . . . . . . . . . . . . . . .	KJ395480
	IINF/PY/00/Chaco23	Chaco, Paraguay	*Triatoma infestans*	. . . . . . . . . . . . . . . .	KJ395481
	IINF/PY/00/T655	Chaco, Paraguay	*Triatoma infestans*	. . . . . . . . . . . . . . . .	KJ395482
TcIV	IINF/AR/00/LHVA	Chaco, Argentina	*Triatoma infestans*	. . . . . . . . . . . . . . . .	KJ395483
TcV	IINF/CL/00/Vinch101	Limari, Chile	*Triatoma infestans*	. . . . . . . . . . . . . . . .	KJ395484
	MHOM/BO/00/92:80	Santa Cruz, Bolivia	*Homo sapiens*	. . . . . . . . . . . . . . . .	KJ395485
	IINF/BR/00/Bug2148	Rio Grande do Sul, Brazil	*Triatoma infestans*	. . . . . . . . . . . . . . . .	KJ395486
	IINF/PY/00/Para6	Paraguari, Paraguay	*Triatoma infestans*	. . . . . . . . . . . A . . A .	KJ395487
TcVI	MHOM/BR/00/CL Brener	Rio Grande do Sul, Brazil	*Triatoma infestans*	. . . . . . . . . . . . . . . .	KJ395488
	MHOM/BO/00/P251	Cochabamba, Bolivia	*Homo sapiens*	. . . . . . . . . . . . . . . .	KJ395489
	IINF/PY/00/Chaco17	Chaco, Paraguay	*Triatoma infestans*	. . . . . . . . . . . . . . . .	KJ395490
	IINF/PY/00/Chaco9	Chaco, Paraguay	*Triatoma infestans*	. . . . . . . . . . . . . . . .	KJ395491
	IINF/AR/00/EPV20-1	Chaco, Argentina	*Triatoma infestans*	. . . . . . . . . . . . . . . .	KJ395492
	IINF/CL/00/VFRA	Francia, Chile	*Triatoma infestans*	. . . . . . . . . . . . . . . .	KJ395493

a. =  no amino acid change.

### Linear B-epitope profiling

Computer analysis of the TSSA-I and the TSSA-II/V/VI common epitope was performed using EpiQuest-B software (v 2.1.17, Matrix B7.1) from Aptum Biologics Ltd (Southampton, Hampshire, UK). The algorithm of the program allows prediction of potential linear B-epitopes and their immunogenicity. The data were used in graphical format.

### Accession numbers

Nucleotide sequences derived in this manuscript are available under GenBank accession numbers KJ395471 - KJ395493.

## Results

### TSSA provides potential epitopes specific for each *T. cruzi* lineage

The structures and sequences of the peptides synthesised, indicating the lineage-specific amino acids, are shown in Figure 1, as based on the comparisons of diversity previously described [24]. In addition to the peptides representing single lineages we synthesised two chimeric peptides, one with TSSA-I residues at the N terminus and TSSA-II residues at the C terminus, and the second with TSSA-II at the N terminus and TSSA-I at the C terminus (Figure 1B).

Consistent with the known extensive genomic divergence between TcI and TcII, eight residues differed between their TSSA potential epitopes. Five and six residues separated TSSA-II from TSSA-III and TSSA-IV, respectively. Four residues distinguished TSSA-I from TSSA-III and two residues separated TSSA-I from TSSA-IV, in accord with their somewhat greater affinity with TcI. A single residue differed between the TSSA-II haplotype shared by TcII, TcV and TcVI and the second haplotype present at the heterozygous locus in the hybrids TcV and TcVI.

### Synthetic peptides are recognised by serum antibodies

Sera from mice experimentally infected with biological clones of TcII, TcV and TcVI strains recognised TSSApep-II/V/VI in serology by ELISA, and sera from TcIII and TcIV murine infections reacted with the corresponding TSSA peptides (Bhattacharyya et al, in preparation), encouraging the evaluation described here of the diagnostic potential of all the synthetic peptides with sera from patients with chronic Chagas disease.

**Figure 2:**
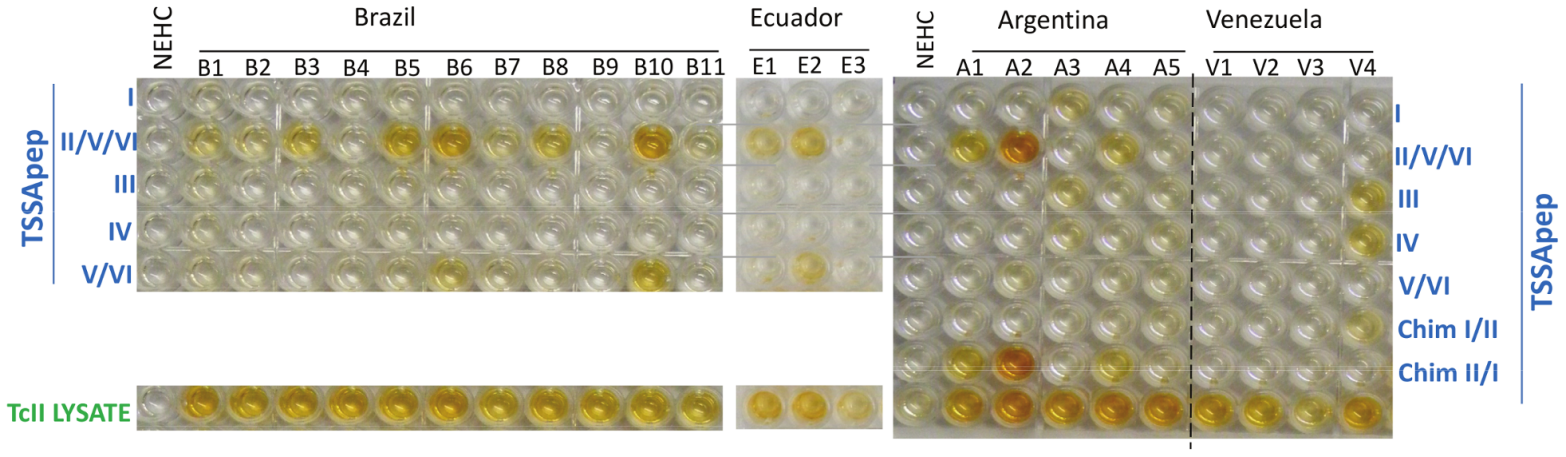
Chagasic sera recognise TSSA lineage-specific peptides. Lineage-specific peptides or lysate were added to rows of the ELISA plate as indicated. ELISA plates showing: recognition of TSSApep-II/V/VI and TSSApep-V/VI, among Brazilian, Ecuadorean and Argentine sera; recognition of TSSApep-I and TSSApep-IV by an Argentine serum and a Venezuelan serum; recognition of chimera TSSApep-II/I by Argentine sera. All patients were seropositive with *T. cruzi* lysate. NEHC =  non-endemic healthy control.

Figure 2 shows examples of ELISA plates with *T. cruzi* lysate and lineage-specific synthetic peptides as antigens. Sera from normal healthy endemic controls did not react with the *T. cruzi* lysate or with any of the synthetic peptides. Without exception all sera from patients with chronic Chagas disease recognised the *T. cruzi* TcII lysate antigen preparation. Figure 2 also provides examples of sera from Brazil, Argentina and Ecuador that recognised TSSApep-II/V/VI, indicative of infection with TcII,TcV or TcVI. A positive result for the epitope derived from the TcV/VI specific haplotype indicates definite infection with TcV or TcVI. Some of these sera (e.g. B6 & B10) reacted with both TSSApep-II/V/VI *and* TSSApep-V/VI representing the haplotype restricted to TcV and TcVI, indicating infection with a hybrid strain, possibly in conjunction with a TcII infection. Recognition of the TcV and TcVI restricted epitope was never seen in the absence of recognition of the TSSApep-II/V/VI.

A Venezuelan serum (V4 in Figure 2) recognised TSSApep-IV, consistent with the known presence of TcIV as a secondary agent of Chagas disease in Venezuela [16]. However, this serum also bound to TSSApep-III, which only differs by 2 of 16 residues.

An antibody response to TSSApep-I was exceptional, only two sera were reactive from the entire set of samples (Table 1) of which one weak reactor (Argentina A3) is shown in Figure 2.

Chimera TSSApep-I/-II and chimera TSSApep-II/-I were designed to determine whether the antigenic epitope resided at the N or C terminus of the peptides. Chimera TSSApep-II/-I was recognised by 71/83 TSSApep-II/V/VI reactive sera, as demonstrated for example by Argentine patients A1, A2 and A4 (Figure 2). In comparison, only 11/83 recognised the chimera TSSApep-I/-II, indicative that, although not precisely mapped, the dominant region of the epitope lies towards the N terminus of the peptide and that in some patients the N terminus is adequate to provide a detectable epitope. A single TSSApep-IV/TSSApep-III positive serum also recognised the chimera TSSApep-I/-II peptide.

Four of 186 samples responded to all wells containing peptides; these were demonstrated to bind non-specifically to avidin in the absence of peptide, but not to cross react with milk proteins (data not shown).

### Rare recognition of the TSSA-II/V/VI common peptide in northern South America

The 186 sera from patients with chronic Chagas disease spanned a geographical range from Argentina to Venezuela. Three Southern Cone countries were included, where TcII, TcV and TcVI have been reported to be endemic, and three countries from northern South America, where TcI is considered to predominate. A summary of the geographical distribution of the antibody responses to all the lineage-specific synthetic peptides is shown in Table 1. Of the sera recognizing TSSApep-II/V/VI, 79 out of 83 were from the Southern Cone countries and four were from Ecuador. Of these 83 sera, 13 sera also recognised TSSApep-V/VI, 12 from Southern Cone countries and one of the four sera from Ecuador, indicating presence of TcV or TcVI, possibly with TcII co-infection. Independently of the lineage-specific peptides, we also examined the response to two different chimera peptides, each comprising different combinations of sequences from TSSApep-I and TSSApep-II/V/VI. Of the Bolivian, Ecuadorean and Argentine sera which reacted with TSSApep-II/V/VI, all reacted with chimera TSSApep-II/-I, but only two samples (Ecuadorean) also reacted with chimera TSSApep-I/-II. In the case of Brazilian samples, of the 67 that reacted with TSSApep-II/V/VI, 55 reacted with chimera TSSApep-II/-I, and of these 55, 9 also reacted with chimera TSSApep-I/-II. Only one sample (Venezuelan) reacted with chimera TSSApep-I/-II but not with TSSApep-II/V/VI or chimera TSSApep-II/-I. TSSApep-I failed to detect antibodies, regardless of origin of the chagasic sera, with the exception of two sera, one each from Brazil and Argentina. Four sera recognised both TSSApep-IV and TSSApep-III, consistent with cross-reaction due to the close similarity between these epitopes.

**Figure 3:**
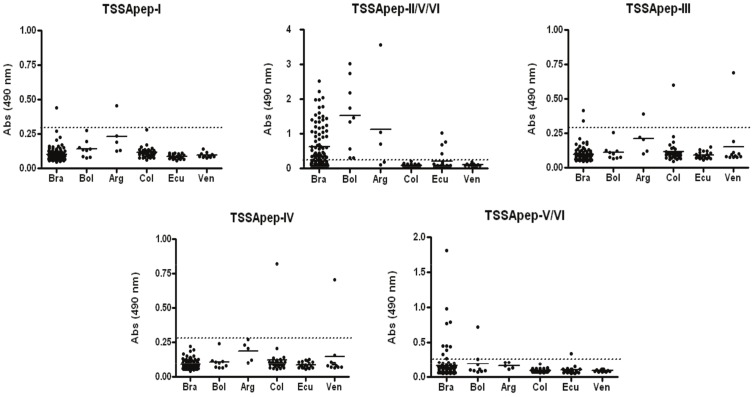
Recognition of TSSA lineage-specific peptides shows a disparate geographical distribution. Each data point represents the mean A_490_ of the reaction of duplicates of each serum sample with the peptides. Means of each country data set are represented by solid horizontal lines; cut-offs, derived from the mean +3SD for each peptide with the EHCs, are shown as dotted line on each graph. Circled and boxed values indicate the same sera. Resolution for the Brazilian responses to TSSApep-II/V/VI is increased by smaller symbols.

The country by country distribution of antibody recognition of the peptides is given in Table 1. ELISA cut-offs and absorbance values for each lineage-specific peptide are shown in Figure 3. Each data point represents the mean A_490_ readout of duplicate assays of the serum sample with the lineage specific peptides. In Figure 3, the samples giving the highest reading for TSSApep-III from Colombia and Venezuela are the same samples that recognised TSSApep-IV.

### Antibodies to the TcII/TcV/TcVI peptide are more frequent among symptomatic Brazilian patients

60/63 of the Brazilian patients with chronic Chagas disease who were seropositive against TSSApep-II/V/VI had detailed clinical evaluation, and of these 60 patients, 23 (38%) had ECG abnormalities typical of Chagas disease. 23/28 patients seronegative for TSSApep-II/V/VI also had detailed clinical evaluation, but in contrast only 4 of these latter, different 23 patients had such ECG abnormalities (p<0.0001).

### Novel bioinformatic algorithms predict highly antigenic residues

**Figure 4:**
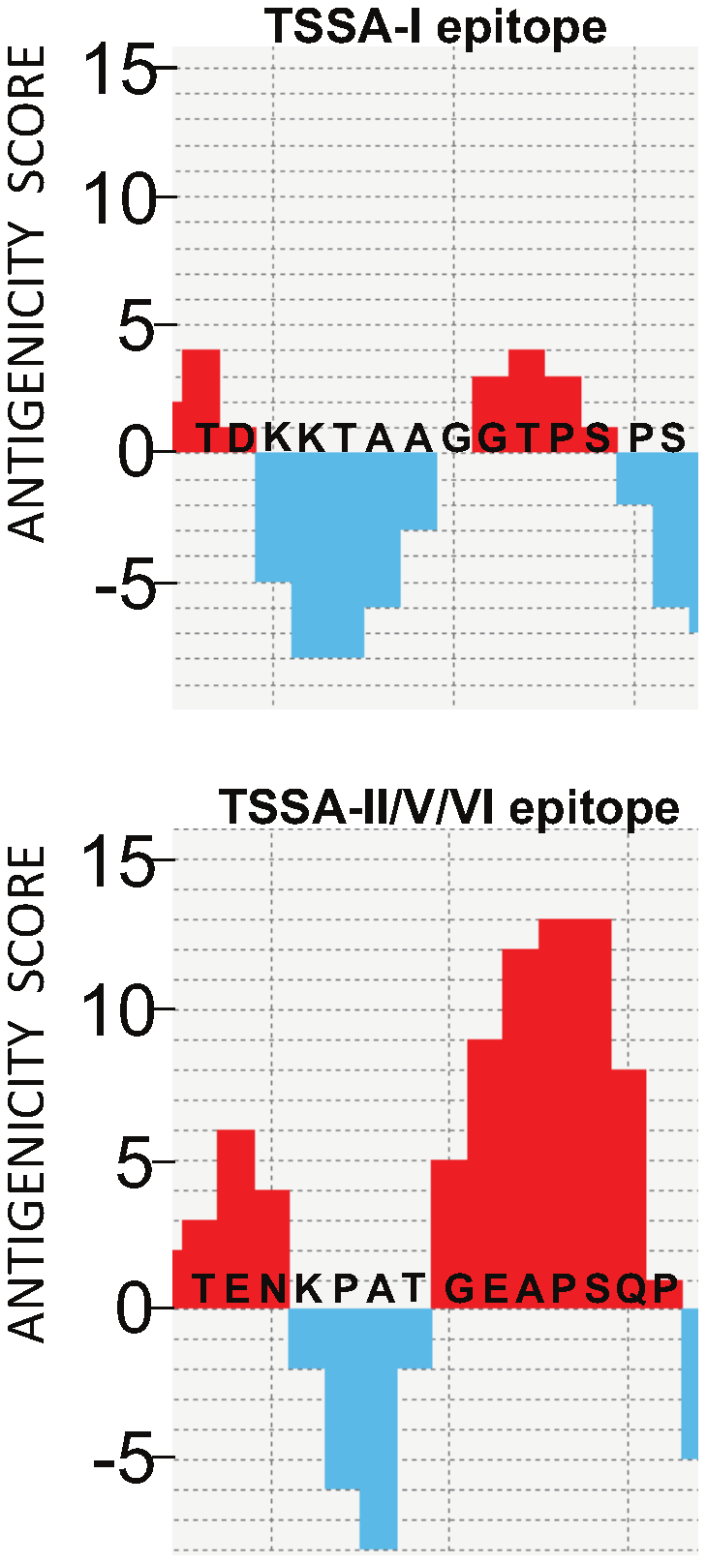
Computer-predicted antigenicity score is much higher for TSSA-II/V/VI sequence than TSSA-I. Polymorphic sequences of [A] TSSA-I and [B] TSSA-II/V/VI showing regions of high antigenicity in red, and low antigenicity in blue. A few amino acid replacements in TSSA-I lead to disappearance of the immunogenic epitope that is present in TSSA-II/V/VI sequence.

The sequences coding for the TSSA proteins containing the TSSApep-I and TSSApep-II/V/VI epitopes were subjected to a novel bioinformatic analysis using EpiQuest-B program that builds the immunogenicity profile for linear protein sequences and predicts the location and potential immunogenicity of the linear B-cell epitopes (Litvinov et al, in preparation) in order to give an antigenicity score for the polymorphic region. The algorithm predicted high scores within the TSSApep-II/V/VI epitope region, but much lower for TSSApep-I, as shown in Figure 4.

The different antigenicity scores indicate that the N-terminal TEN in TSSApep-II/V/VI is the dominant epitope, explaining the frequent recognition of chimera TSSApep-II/-I (Figure 1) despite the higher scoring C-terminal GEAPS, which provides a secondary site of recognition for some (Brazilian) sera that respond to chimera TSSApep-I/-II (Figure 1). Neither the TEN nor GEAPS is present in TSSApep-I, explaining the rare recognition of this epitope. The GEAPS, which is also present in TSSApep-III and TSSApep-IV, gave much lower antigenicity scores in these peptides, in the absence of the upstream TEN in these sequences (data not shown).

### Comparative diversity of the putative TcI-applicable peptide reveals high conservation across lineages

**Figure 5:**
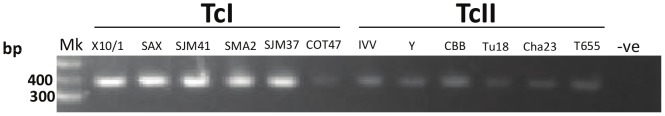
PCR amplification of the ORF containing the reported TcI-applicable epitope. Only the amplicon of predicted size (381 bp) was amplified by the reaction conditions. –ve =  no template DNA control. Mk =  Hyperladder IV (Bioline, UK).

Mendes et al [26] used heterozygous loci in the CL Brener genome to identify candidate lineage-specific epitopes. One conserved and three potentially lineage-specific peptides were synthesised, described as deriving from hypothetical protein Tc00.1047053510421.310 (conserved epitope peptide), putative RNA-binding protein Tc00.1047053511837.129 (for TcI serology), putative ADP-ribosylation factor GTPase activating protein Tc00.1047053511589.70 (for TcII), and putative DNA-directed RNA polymerase III subunit Tc00.1047053510359.320 (for TcVI) that displayed some discriminatory power in ELISAs and affinity-ELISAs based on differential range of absorbance values. The putative TcI epitope was described as restricted to TcI and TcVI and applicable to serological identification of a specific response to TcI. We examined the diversity in the coding sequence for this epitope, using a panel of *T. cruzi* strains across the lineages (Table 2). A single amplicon of 381bp was produced by PCR from all strains using primers MenTcI FOR and MenTcI REV. Examples from TcI and TcII strains are shown in Figure 5.

However, in comparative sequencing across isolates representing the lineages we found this epitope to be highly conserved (Table 2). This epitope had the same amino acid sequence across all the strains and lineages analysed here with the exception of strains Y and CBB (TcII) and Para 6 (TcV). In strains Y and CBB, a heterozygous nucleotide (G/C) residue led to the presence of two predicted amino acids, Ser or Thr.

The region homologous to the reported TcI-applicable epitope, which was described as specific to a TcII strain, but given only as amino acid sequence [26], was subject to BLAST against NCBI and TriTrypDB databases. There were very low stringency homologies returned by TriTryp BLAST to various *T. cruzi* proteins (mainly around the PPP tripeptide), none of which was described as RNA-binding proteins. On NCBI BLAST, highest-scoring matches were to various bacteria and fungi, none to trypanosomes. Furthermore, none of 55 sera from northern countries of South America, where TcI is highly endemic, bound to the TcI synthetic peptide reported in Mendes et al in our ELISA assays (data not shown).

## Discussion

Kong et al [32] developed lineage-specific serology for the protozoan parasite *Toxoplasma gondii*, which is difficult to isolate from chronically infected patients, and most isolates of which are classified into clonal lineages type I, II, or III. Serology with synthetic peptides based on diversity within the dense granule proteins GRA6 and GRA7 was able to distinguish type II from non-type II infections in humans. Using discriminatory serology, it was possible to demonstrate that the *T. gondii* lineages had different continental distributions [33], and that adult offspring of type I-infected mothers had a significantly increased risk for the development of psychoses [34].

Here we have used detailed comparative analysis of the genetic diversity of the *T. cruzi TSSA* gene encoding the protein core of the mucin TSSA, to design synthetic peptides for lineage-specific serology of *T. cruzi* infection history. These epitopes were presented on an avidin-coated solid phase via an amino terminal biotin-label linked to a polyethylene glycol-glycine spacer to increase rotation and ensure that each amino acid side chain could freely interact with antibodies. We synthesised and tested these lineage-specific peptides in ELISA with 186 human sera from six countries, three in the Southern Cone region of South America and three in northern South America. We have selected these countries because typing of *T. cruzi* isolates with multilocus enzyme electrophoresis (MLEE) [6], multilocus sequence typing (MLST) [35] and multilocus microsatellite typing (MLMT) [17] has repeatedly indicated the predominance of TcII, TcV and TcVI as the agents of Chagas disease in Southern Cone countries yet the contrasting high prevalence of TcI in patients North of the Amazon [7], [11]. Nevertheless, there have been some reports that TcII, as identified by genotyping, can be found among isolates from humans and domestic triatomine bugs in northern endemic regions, for example in Colombia and Guatemala [36]–[38].

Lineage-specific serology is therefore of special interest for *T. cruzi,* because of the disparate geographical distributions of both the *T. cruzi* lineages and clinical manifestations of chronic Chagas disease. Thus as long ago as 1981, Miles et al proposed that the presence of chagasic cardiomyopathy with megaoesophagus and megacolon in Southern Cone countries, yet apparent absence of associated megasyndromes from Venezuela, may be related to the comparative predominance of TcI as the agent of Chagas disease in northern South America [6]. Nevertheless the evidence of a link between infecting lineage of *T. cruzi* and prognosis of chronic Chagas disease remains circumstantial. As with *T. gondii*, this is partly due to the difficulty of isolating and genotyping *T. cruzi* from chronic chagasic patients. Blood culture and xenodiagnosis have limited sensitivity and may be selective for faster growing biological clones. Furthermore, even if parasites or DNA can be recovered from chronically infected patients, the resultant *T. cruzi* isolates may not be representative of the genetic diversity in the patient, because *T. cruzi* replicates intracellularly and lineage genotypes may be sequestered in the tissues but not recoverable from the circulating blood [14]. Serology with lineage-specific antigens provides a means of profiling an individual's history of *T. cruzi* infection, to overcome inaccessibility of the parasite to direct genotyping during chronic infections.

TSSA provides a good candidate for development of synthetic peptide-based, lineage-specific serology, because no TSSA homologue beyond the species *T. cruzi* has been detected by genomic comparisons, and a lineage-specific candidate epitope can be represented by a single synthetic peptide. Thus such peptides are unlikely to generate false positive ELISA results with sera from endemic healthy controls or from patients with other infectious or autoimmune diseases. In the multiple ELISAs performed here none of the healthy controls recognised any of the synthetic peptides, and all were also serologically negative with the *T. cruzi* lysate (Figure 2). However, sera from four of the chagasic patients bound non-specifically to plates coated with avidin alone and thus spuriously appeared to recognise all peptides; such artifactual binding to avidin has been observed in other serological studies [39].

Since the initial report of the sequence and antigenic dimorphism of TSSA by Di Noia et al [15], *E. coli*-produced recombinant TSSA proteins have been used as antigen with human and animal sera, as summarised in Table 3. Recognition of only TSSA-II by chronic chagasic sera from the Southern Cone region was initially interpreted as suggesting that only TcII caused chronic Chagas disease [15]. However there are many descriptions of Chagas disease and chronic chagasic cardiomyopathy in TcI endemic regions. Recognition of recombinant TSSA-I by human chagasic sera has been reported by western blot but not by ELISA [18], [21]. One western blot study with recombinant TSSA-II and TSSA-I has recorded an unexpected level of TcII in northern South America and Mexico [21]. The recombinant TSSA proteins used as antigens as described encompass up to 26 amino acids flanking the polymorphic region [15], [20], [21], which are highly conserved between TSSA-I and TSSA-II.

**Table 3:** Reports of TSSA recombinant proteins in serological assays.

Reference	rTSSA Tc lineage	Assay	Sources of human sera	Authors' reports
[15]	I, II	ELISA, CL-ELISA^a^	Argentina, Brazil, Chile	TSSA dimorphism; chagasic sera only recognise rTSSA-II; TcI or TcII -infected animal sera recognised the homologous rTSSA form, without cross-reactivity.
[18]	I, II/V/VI	Western blot	Argentina	TcII/V/VI and TcI co-infection in cases of chagasic cardiomyopathy; TcII/V/VI also in indeterminate clinical form.
[19]	II	ELISA	Argentina	rTSSA-II recognised by chagasic but not non-chagasic or cutaneous leishmaniasis sera. rTSSA-II recognised by canine sera from TcVI but not TcI or TcIII infections.
[20]	II^b^	CL-ELISA a	Brazil	rTSSA-II 98% sensitive; no response to rTSSA-I; minimal cross-reactivity with *Leishmania* sera.
[21]	I, II	Western blot	Argentina, Colombia, Mexico, Paraguay, Venezuela	Recognition of TSSA-II, TSSA-I and TSSA-II/I in northern South America and Mexico; almost exclusively rTSSA-II in southern South America.
[22]	I, II/V/VI	Western blot, ELISA	Argentina, Bolivia, Paraguay	TcII/V/VI predominant in pregnant chagasic women; no recognition of TSSA-I reported.
[23]	II^b^	ELISA	Argentina	TcII and/or TcV/TcVI in the north of Salta province.

a Chemiluminescent ELISA; ^b^called by authors TSSA VI, but the same as that first described as TSSA-II.

The lineage-specific peptide representing the epitope common to TcII/TcV/TcVI was recognized by a large number of sera from Brazil; a proportion of these sera also bound to TSSApep-V/VI. All duplicate separate samples from the same patients gave indistinguishable results. The Brazilian sera tested here originated from the states of Goiás and Minas Gerais, where TcII human infections are known to be prevalent, TcV and TcVI are also present and TcI is (relatively) uncommon [11], [40], [41], although TcI is well represented among Brazilian sylvatic transmission cycles [41], [42]. However, a substantial minority of the Brazilian serum samples (31/98 (31.6%)) did not react with TSSApep-II/V/VI. Thus sensitivity of the TSSApep-II/V/VI ELISA does not appear to be absolute for TcII/TcV/TcVI *T. cruzi* infections (Figure 2, Table 1). It is possible that corresponding antibodies in the TcII/TcV/TcVI seronegative patients were simply below the threshold for detection in the ELISA, although this seems unlikely because such patients remained equally seronegative against the peptides even when re-tested at the higher serum concentration of 1∶100 (data not shown). Alternatively, some patients may fail to generate an immune response to the epitope or there may be as yet undiscovered TSSA diversity in some *T. cruzi* TcII strains.

Elsewhere in the Southern Cone countries 12 of 15 sera from Bolivia or Argentina were seropositive with TSSApep-II/V/VI, in accord with the known high prevalence of these lineages in those countries [23]. All sera from Bolivia, Argentina and Ecuador, and the great majority of those from Brazil, that recognised TSSApep-II/V/VI also reacted with chimera TSSApep-II/-I indicating that crucial residues reside in the N-terminal part of the TSSA-II/V/VI epitope.

We found that few serum samples from the three countries in northern South America recognized TSSApep-II/V/VI or TSSApep-V/VI. This is consistent with the literature on the geographical distribution of *T. cruzi* lineages based on genotyping of isolates from domestic and sylvatic transmission cycles. In fact only 4 sera from Ecuador were seropositive with TSSApep-II/V/VI out of 66 from these northern countries. At least 3 of these 4 Ecuadorian serum samples originated from the Loja region in southern Ecuador, where TcI has been isolated [43], close to the border with Peru. Risso et al [21] reported the identification of TcII in Colombia, Venezuela, and Mexico using western blots with TSSA-II recombinant antigen. However, when the same Colombian sera samples were tested here using the lineage-specific peptides we found no TSSApep-II/V/VI seropositive patients. Thus with our data we are unable to confirm the presence of TcII/TcV/TcVI in those Colombian patients.

Only four sera, including one from Venezuela where TcIV is known to sporadically infect humans, recognised TSSApep-IV. All four sera also recognised TSSApep-III, which shares 14 of 16 residues, presumably due to cross reaction, as we have observed with experimental murine sera (Bhattacharyya et al, in preparation).

Apart from one Argentine and one Brazilian serum, no clear specific reaction with TSSApep-I was observed, even with sera from known TcI endemic regions in Venezuela, Colombia and Ecuador. The few TSSApep-II/V/VI seropositive samples from Brazil that also reacted with chimera TSSApep-I/-II did not react with TSSApep-I. One possibility is that the TSSA-I protein, if expressed at all in chagasic patients, is not sufficiently immunogenic to generate an antibody response, possibly due to post-translational glycosylation of the core peptide sequence. Identification of the disaccharide Galα(1,3)Galβ as the immunodominant glycotope present in the O-linked mucins, i.e., those glycosylated on serine or threonine residues of the peptide chain, has been reported recently [44], [45], and both serine and threonine are represented by one additional residue in TSSApep-I as compared with the TSSA-II epitope. However, equally likely, the TSSA I epitope may be conformational, with a structure that is not represented by the linear peptide. Also, alternative immunodominant epitopes elsewhere in TSSA-I may skew the humoral response away from the sequence represented by TSSApep-I.

We were interested to see whether there was a difference in the proportions of TSSApep-II/V/VI seropositive and seronegative patients presenting with clinical symptoms of chronic Chagas disease. Remarkably, there was a clear statistically significant difference: 23/60 (38%) of the Brazilian TSSApep-II/V/VI seropositives had ECG abnormalities typical of Chagas disease, whereas such abnormalities only occurred in 4/23 (17%) of the seronegatives (p<0.0001). One possible interpretation of these data is that TSSApep-II/V/VI seronegative patients may not be infected with these lineages but with some less pathogenic strains. Alternatively, such seronegative patients may be infected with TcII, TcV or TcVI but the absence of an immune response to the TSSA-II/V/VI common epitope may be an indicator of a long term better prognosis; however confirmation would require a more extensive and longitudinal study. However, the frequencies of megaoesophagus (43% vs 48%) and megacolon (10% vs 8.7%) were not significantly different between the TSSApep-II/V/VI seropositive and seronegative groups respectively.

*Trypanosoma rangeli* is non-pathogenic to humans, is found sympatric with *T. cruzi*, particularly in northern South America, and serological cross-reaction between these species has been recognised [46]. The divergence of the TSSA epitopes in *T. cruzi* and the lack of response to the peptides with sera from northern South America, indicate that monospecific sera from patients infected with *T. rangeli* alone will not recognise these synthetic peptide epitopes.

A recent paper reported the identification of a TcI epitope for lineage-specific serology [26]. However, upon analysing the sequence diversity in the ORF coding for the parent protein across *T. cruzi* lineages, in contrast we found a very high degree of sequence conservation across the lineages. Thus, we were not able to confirm any TcI-specificity of that peptide epitope.

We have demonstrated that synthetic peptides are able to provide *T. cruzi* antigens for lineage-specific serological diagnosis in chronic Chagas disease. Synthetic-peptide based lineage-specific serology has also confirmed the disparate geographical distribution of TcII/TcV/TcVI but found fewer TcII infections in northern South America than reported with western blots and recombinant TSSA-II. Further comparisons of recombinant TSSA antigens and synthetic peptides are indicated. More attempts should be made to design a TcI specific peptide, and by comparative genomics to seek alternative antigens to TSSA that may be lineage-specific. However, such *in silico* methods will need to incorporate structural analysis, and if necessary devise linear peptides that represent conformational epitopes. In a region of Brazil endemic for TcII we find a higher rate of ECG abnormalities among patients with TSSApep-II/V/VI seropositivity than among seronegative patients. Synthetic-peptide antigens clearly have substantial and versatile potential in studying the relationship between a patient's history of infection and clinical status, and they may provide clinical biomarkers for prognosis of Chagas disease.
